# Band-selective universal 90° and 180° rotation pulses covering the aliphatic carbon chemical shift range for triple resonance experiments on 1.2 GHz spectrometers

**DOI:** 10.1007/s10858-022-00404-1

**Published:** 2022-11-24

**Authors:** Stella Slad, Wolfgang Bermel, Rainer Kümmerle, Daniel Mathieu, Burkhard Luy

**Affiliations:** 1 Institute of Organic Chemistry and Institute for Biological Interfaces 4 - Magnetic Resonance, Karlsruhe Institute of Technology (KIT), Fritz-Haber-Weg 6, 76131 Karlsruhe, Germany; 2 Bruker BioSpin GmbH, Rudolf-Plank-Str. 23, 76275 Ettlingen, Germany; 3 Bruker BioSpin AG, Industriestr. 26, 8117 Fällanden, Switzerland

**Keywords:** Triple resonance experiments, 1.2 GHz, Band-selective pulses, Universal rotations, Excitation, refocusing

## Abstract

Biomolecular NMR spectroscopy requires large magnetic field strengths for high spectral resolution. Today’s highest fields comprise proton Larmor frequencies of 1.2 GHz and even larger field strengths are to be expected in the future. In protein triple resonance experiments, various carbon bandwidths need to be excited by selective pulses including the large aliphatic chemical shift range. When the spectrometer field strength is increased, the length of these pulses has to be decreased by the same factor, resulting in higher rf-amplitudes being necessary in order to cover the required frequency region. Currently available band-selective pulses like Q3/Q5 excite a narrow bandwidth compared to the necessary rf-amplitude. Because the maximum rf-power allowed in probeheads is limited, none of the selective universal rotation pulses reported so far is able to cover the full $^{13}$C aliphatic region on 1.2 GHz spectrometers. In this work, we present band-selective 90° and 180° universal rotation pulses (SURBOP90 and SURBOP180) that have a higher ratio of selective bandwidth to maximum rf-amplitude than standard pulses. Simulations show that these pulses perform better than standard pulses, e. g. Q3/Q5, especially when rf-inhomogeneity is taken into account. The theoretical and experimental performance is demonstrated in offset profiles and by implementing the SURBOP pulses in an HNCACB experiment at 1.2 GHz.

**Supplementary Information:**

The online version contains supplementary material available at 10.1007/s10858-022-00404-1.

## Introduction

Modern biomolecular NMR spectroscopy involves high resolution NMR spectroscopy at highest available magnetic fields approaching 1.2 GHz proton resonance frequencies. Such high frequencies impose the requirement for large bandwidths to be covered while the accessible radio-frequency (rf) amplitudes of available probeheads do not increase proportionally. While effective broadband solutions for the coverage of corresponding carbon and nitrogen chemical shift ranges exist (Skinner et al. 2003, 2004; Kobzar et al. 2004; Skinner et al. 2005, 2006; Gershenzon et al. 2007, 2008; Kobzar et al. 2008; Skinner et al. 2012a, b; Ehni and Luy 2012; Nimbalkar et al. 2013; Ehni and Luy 2013), problems arise in protein triple resonance experiments when large carbon bandwidths need to be excited selectively  (Sattler et al. 1999). Commonly applied state-of-the-art band-selective pulses used for carbon spins are G3/G4 (Emsley and Bodenhausen 1990), Q3/Q5 (Emsley and Bodenhausen 1992), Q3_SURBOP/ Q5_SEBOP (Gershenzon et al. 2009), EBURP/IBURP/REBURP (Geen and Freeman 1991), and the Bloch–Siegert-corrected GOODCOP/BADCOP (Coote et al. 2018) pulse shapes. They are all optimized to cover a relatively narrow bandwidth, assuming that larger bandwidths can be simply covered by scaling pulse lengths and rf-amplitudes accordingly. This is certainly the case for magnetic field strengths of most commonly used spectrometers with proton Larmor frequencies of 600–900 MHz. However, the development of magnets with higher and higher fields for further enhanced resolution puts a limitation to pulse scaling: In order to invert the full $^{13}$C aliphatic region on 1.2 GHz spectrometers, Q3 pulses with lowest power requirements would have to be scaled to approximately 26 kHz rf-amplitude. This is higher than the recommended peak rf-amplitude of 25 kHz for a 3 mm TCI CryoProbe. The situation for highly desired 5 mm triple resonance probeheads will be even worse. In addition, other pulse shapes, for example REBURP shapes as the preferred band-selective pulses for INEPT-type transfer elements (Morris and Freeman 1979; Burum and Ernst 1980; Brutscher and Solyom 2017; Haller et al. 2019; Bodor et al. 2020) require much higher rf-amplitudes for the desired bandwidth.

In fact, none of the available selective universal rotation pulses is able to excite or refocus the full $^{13}$C aliphatic region at 1.2 GHz with generally available rf-amplitudes of e.g. 15 kHz. This is even more important as the known salt dependence of available pulse lengths is getting more and more pronounced also on carbon for spectrometers operating at proton Larmor frequencies beyond 1 GHz. We therefore looked into the design of corresponding band-selective shaped pulses based on a modified version of the optimal control-derived GRadient Ascent Pulse Engineering (GRAPE) algorithm (Khaneja et al. 2005).

After a description of the specific algorithm used, resulting band-selective universal 90° and 180° pulse shapes, called SURBOP90 and SURBOP180, are characterized in detail and compared to most frequently used Q5/Q3 and G4 pulses in theory and experiment.

## Theory

### Optimization goal

**Fig. 1:**
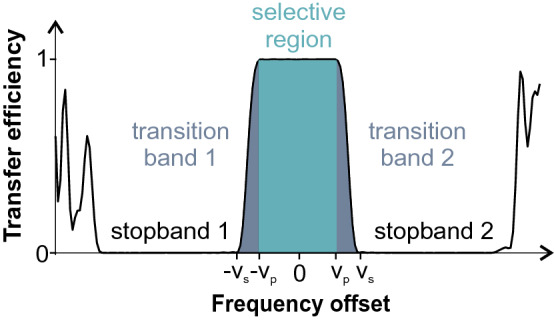
Specified offset regions: For the band-selective SURBOPs five individual areas have been specified: the central selective band to be optimized using the cost function $\varPhi _{sel}$ and defined by the range $\pm \nu _p$, two non-optimized transition regions (frequencies lie between $-\nu _s$ and $-\nu _p$ or $\nu _p$ and $\nu _s$), and two stopbands without effective transverse plane rotations as optimized by $\varPhi _0$. Outside these regions the pulse behaviour is not specified like in the transition region

A protein $^{13}$C spectrum consists of several separate regions that are frequently treated selectively using band-selective pulses: Carbonyl resonances are found at 170–180 ppm, aromatic resonances at 110–140 ppm and aliphatic $^{13}$C resonances with the largest bandwidth are typically in the range of 10–75 ppm. However, to reach also less frequently occurring extreme values for aliphatic resonances, a selective coverage of roughly 80 ppm is desirable (Sattler et al. 1999). The main goal was therefore the design of band-selective 90° and 180° pulses covering 80 ppm at a $^{13}$C Larmor frequency of 300 MHz, corresponding to 24,000 Hz or $\nu _p=12{,}000$ Hz as defined in Fig. 1. As an additional condition we imposed that aromatic and carbonyl resonances should not be affected by the pulses. This leads to a transition region of approximately 20 ppm, or $\nu _{\text{s}}-\nu _{\text{p}}=6000$ Hz. Although in principle a single stopband left from the selective region is sufficient, two symmetric stopbands have been specified, in which the shaped pulse does not lead to any effective rotations in the *x*,*y*-plane. This way each pulse shape is more generally applicable and also tolerates time or phase reversal, which otherwise would lead to inverted stopband frequencies. To include most potential carbon spins, the bandwidth of each of the stopbands was chosen to be 140 ppm or 42,000 Hz, respectively.

Additional conditions concern acceptable pulse lengths, rf-amplitudes and the types of pulses to be optimized. The pulse lengths should not exceed the lengths of commonly used aliphatic selective pulses at moderate field strengths of 600–800 MHz proton Larmor frequencies. We therefore decided to limit pulse lengths to 400 ${\upmu }$s. Maximum rf-amplitudes, on the other hand, should be viable for all types of triple resonance probeheads, including a certain buffer for samples with high salt concentrations. We therefore opted for an rf-amplitude limitation of 15 kHz.

For the detailed implementation of the GRAPE algorithm the pulse type had to be specified. There are two major types of pulses that are discriminated in pulse engineering: point-to-point (PP) and universal rotation (UR) pulses. A pulse of the first type can only transfer one initial magnetization component to a specific target component. For example, *z*-magnetization can be transformed to *x*-magnetization. However, the final orientation of vectors that are initially orthogonal to *z* can be anywhere on the *yz*-plane. Conversely, UR pulses rotate an initial magnetization vector by a defined rotation angle and axis, independent of the inital magnetization state. As such, PP pulses are limited in their application, while UR pulses can generally be used as corresponding hard pulses for the selected bandwidth. As systematic studies of broadband UR pulses (Kobzar et al. 2012) show that the specified pulse lengths should be sufficient for universal rotations, we decided to implement the GRAPE algorithm for the design of Selective Universal Rotations by Optimized Pulses (SURBOP).

### Optimization procedure

#### Univeral rotations and quaternions

Optimal control based algorithms allow simultaneous optimization of hundreds and even thousands of parameters and therefore are very powerful tools for pulse optimization. Here, we want to summarize the specific aspects and the general workflow of the GRAPE-derived algorithm used for optimizing the SURBOP90 and SURBOP180 pulses.

A shaped pulse can be seen as a sequence of *N* short pulses of length $\varDelta t$ with piecewise constant rf-amplitudes, where the $k{\text {th}}$ pulse results in the two controls ${\text{u}}_{x,k}$ and $\mathrm {u}_{y,k}$ that represent the *x* and *y*-components of the shaped pulse. The goal of the optimization is to find values of these controls that minimize the differences between the desired target propagator of the pulse, ${\text {U}}_F$, and the final propagator obtained with the current control amplitudes, ${\text {U}}_N$. Following variational theory and the Lagrange formalism, it is possible to initialize the algorithm with arbitrary values and to calculate gradients towards guarenteed better performance in every iteration step of the optimal control algorithm until a local performance optimum is reached.

Since the aim of the optimization is to obtain universal rotation pulses, the adequate description of the problem is the use of Caley–Klein parameters (Cayley 1859; Klein 1871, 1873) or the equivalent formulation using quaternions (Blümich and Spiess 1985; Emsley and Bodenhausen 1992). The theory of the quaternion formalism has been explained in the cited literature. Therefore, we will only provide most important relations that are required for understanding the algorithm used.

A target propagator ${\text {U}}_F$ as well as the $k{\text {th}}$ propagator ${\text {U}}_k$ of a pulse shape applied to an ensemble of non-interacting spins can be written as a vector of four quaternion elements

1 $$\begin{aligned} {\text {U}}_{F}= \begin{pmatrix} {\text {A}}_{F} \\ {\text {B}}_{F} \\ {\text {C}}_{F} \\ {\text {D}}_{F} \end{pmatrix}; \ \ \ \ {\text {U}}_{k}= \begin{pmatrix} {\text {A}}_{k} \\ {\text {B}}_{k} \\ {\text {C}}_{k} \\ {\text {D}}_{k} \end{pmatrix}, \end{aligned}$$

where the quaternion elements of the propagator in step *k* can be derived from the control amplitudes (${\text {u}}_{x,k}$ and ${\text {u}}_{y,k}$) and the frequency offset of the spin $\nu _{\text {off}}$ by

2 $$\begin{aligned} {\text {A}}_{k}&={\text {n}}_{x,k}\sin \Big (\frac{\theta _k}{2}\Big ) \nonumber \\ {\text {B}}_{k}&={\text {n}}_{y,k}\sin \Big (\frac{\theta _k}{2}\Big ) \nonumber \\ {\text {C}}_{k}&={\text {n}}_{z,k}\sin \Big (\frac{\theta _k}{2}\Big ) \nonumber \\ {\text {D}}_{k}&=\cos \Big (\frac{\theta _k}{2}\Big ) \end{aligned}$$

with the unit vectors of the effective rotation axis

3 $$\begin{aligned} {\text {n}}_{x,k}&=2\pi \varDelta t {\text {u}}_{x,k}/\theta _k, \nonumber \\ {\text {n}}_{y,k}&=2\pi \varDelta t {\text {u}}_{y,k}/\theta _k, \nonumber \\ {\text {n}}_{z,k}&=2\pi \varDelta t \nu _{\text {off}}/\theta _k, \end{aligned}$$

and the overall rotation angle $\theta _k$ in timestep *k*, defined as

4 $$\begin{aligned} \theta _k =2\pi \varDelta t\sqrt{{\text {u}}_{x,k}^2+{\text {u}}_{y,k}^2+\nu _{\text {off}}^2}. \end{aligned}$$

For example, the quaternion representations of effective $90^{\circ }_x$ and $180^{\circ }_x$ rotations are given by

5 $$\begin{aligned} {\text {U}}_{90_x}= \begin{pmatrix} \frac{1}{\sqrt{2}} \\ 0 \\ 0 \\ \frac{1}{\sqrt{2}} \end{pmatrix}; \ \ \ \ {\text {U}}_{180_x}= \begin{pmatrix} 1 \\ 0 \\ 0 \\ 0 \end{pmatrix} \end{aligned}$$

The properties of quaternions allow a straightforward composition of a cumulative effective rotation ${\text {X}}_{2}$ out of two successive rotations ${\text {U}}_{2}$ and ${\text {U}}_{1}$: In the first step, a rotation matrix ${\text {R}}_{2}$ is composed using the quaternion elements of ${\text {U}}_{2}$. In the second step, the composite rotation ${\text {X}}_{2}$ can be calculated according to

6 $$\begin{aligned} {\text {X}}_{2}={\text {R}}_{2}{\text {U}}_{1}= \begin{pmatrix} {\text {A}}_{X_2} \\ {\text {B}}_{X_2} \\ {\text {C}}_{X_2} \\ {\text {D}}_{X_2} \end{pmatrix}= \begin{pmatrix} +{\text {D}}_{2} &{} -{\text {C}}_{2} &{} +{\text {B}}_{2} &{} +A_2 \\ +{\text {C}}_{2} &{} +{\text {D}}_{2} &{} -A_2 &{} +{\text {B}}_{2} \\ -{\text {B}}_{2} &{} +A_2 &{} +{\text {D}}_{2} &{} +{\text {C}}_{2} \\ -A_2 &{} -{\text {B}}_{2} &{} -{\text {C}}_{2} &{} +{\text {D}}_{2} \end{pmatrix} \begin{pmatrix} {\text {A}}_{1} \\ {\text {B}}_{1} \\ {\text {C}}_{1} \\ {\text {D}}_{1} \end{pmatrix} \end{aligned}$$

Using this formalism, the overall pulse propagator at timestep *k* can be calculated as follows, when the control amplitudes of the pulse are known:

7 $$\begin{aligned} {\text {U}}_{k}={\text {X}}_{k}={\text {R}}_k{\text {R}}_{k-1}\ldots {\text {R}}_1{\text {U}}_0 \end{aligned}$$

with $${\text {U}}_0= \begin{pmatrix} 0 \\ 0 \\ 0 \\ 1 \end{pmatrix}$$.

#### Cost functions for SURBOP pulses

A large variety of pulses has been optimized using GRAPE-derived algorithms (Skinner et al. 2003, 2004; Kobzar et al. 2004; Skinner et al. 2005, 2006; Gershenzon et al. 2007, 2008; Kobzar et al. 2008; Skinner et al. 2012a, b; Ehni and Luy 2012; Nimbalkar et al. 2013; Ehni and Luy 2013; Goodwin et al. 2020; Ehni et al. 2022; Haller et al. 2022), including UR pulses (Skinner et al. 2012a; Kobzar et al. 2012). However, the cost function originally introduced in the GRAPE publication (Khaneja et al. 2005) $\varPhi _{UR}=\langle \ {\text {U}}_F| {\text {X}}_N \rangle$ cannot be used to optimize a desired stopband behaviour. The algorithm therefore had to be modified for band-selective pulses. Based on the idea originally introduced to optimize individual components of PP pulses (Skinner et al. 2005), the component-specific difference between the desired and actual overall propagator is minimized. Interestingly, the same concept for cost functions were used for the design of Q5/Q3 pulses (Emsley and Bodenhausen 1992), while the algorithm used back then was very different from ours. The cost function for a specific isochromat *i* in the passband is then defined as

8 $$\begin{aligned} \begin{aligned} \varPhi _{\text {sel},i}=&({\text {A}}_{F}-{\text {A}}_{X_N,i})^2 + ({\text {B}}_{F}-{\text {B}}_{X_N,i})^2 \\&+ ({\text {C}}_{F}-{\text {C}}_{X_N,i})^2 + ({\text {D}}_{F}-{\text {D}}_{X_N,i})^2 \end{aligned} \end{aligned}$$

These values are then multiplied with the weighting factor $w_{sel}$ and added up to obtain the effective additive cost of the selective region

9 $$\begin{aligned} \varPhi _{\text {sel}}= w_{\text {sel}} \sum _{i=1}^n \varPhi _{\text {sel},i}. \end{aligned}$$

The cost function for isochromats *j* in the stopbands is different, because here the only two conditions are that the net rotation angles over the *x*- and *y*-axes equal zero, while arbitrary *z*-rotations, eventually leading to a Bloch–Siegert shift, are allowed. Therefore, the components ${\text {C}}$ and ${\text {D}}$ are neglected, allowing maximum freedom for the optimization. The summation over all *j* in the stopband results then in the stopband cost function

10 $$\begin{aligned} \varPhi _0= w_0 \sum _{j=1}^m ({\text {A}}_{X_N,j}-0)^2+ ({\text {B}}_{X_N,j}-0)^2 \end{aligned}$$

An alternative approach, involving a point-to-point constraint, could also be used in order to optimize the behaviour in the stopbands. However, it is computationally costly to combine cost functions of UR- and PP-type in the same optimization. Therefore, we chose to implement the cost function described in the equation above.

The global cost function to be minimized is then calculated as the overall additive cost

11 $$\begin{aligned} \varPhi =\varPhi _0+\varPhi _{{\text {sel}}} \end{aligned}$$

**Fig. 2:**
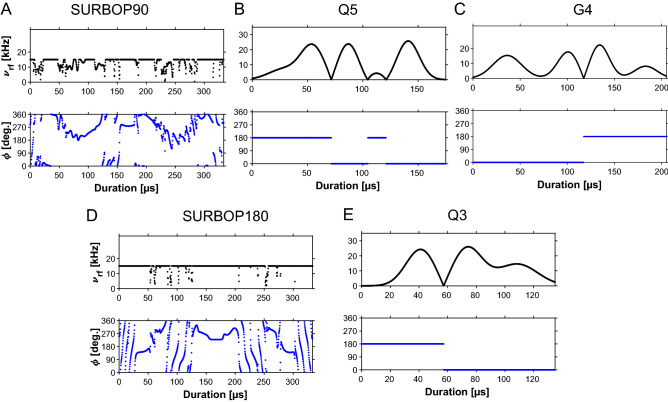
Shapes of the SURBOP pulses compared to Q5/Q3 pulses: Compared to the standard Q5/G4/Q3 pulses (B, C, E), the newly derived SURBOP pulses (A, D) show a very different amplitude and phase behaviour. During SURBOP90 the peak rf-amplitude is reached about twenty times and often maintained for several microseconds. Between these areas, the rf-amplitude drops very briefly to about half of the maximum rf-amplitude. In contrast, Q5 and G4 reach values close to their peak rf-amplitudes only three times and Q3 only twice. Note that the peak rf-amplitude is 26.0 kHz for Q5/Q3 and 22.7 kHz for G4, while SURBOP pulses with a peak rf-amplitude of 15 kHz cover the same selective region. The phase modulations of SURBOP pulses are predominantly smooth. In contrast, the phase of all standard pulses remains constant for extended periods of time, but with a few abrupt phase changes of 180$^{\circ }$

The local gradients for particular offsets *i* or *j* and individual pulses *k* can be calculated by the derivatives $\partial \varPhi /\partial u_{x,k}$ and $\partial \varPhi /\partial u_{y,k}$ of the global cost function. Using the solution provided by Khaneja et al. (2005), as a first step the effective propagators ${\text {X}}_{k}={\text {R}}_k{\text {R}}_{k-1}\ldots {\text {R}}_1{\text {U}}_0$ for all $k\le N$ are calculated. Then, the costate propagators ${\text {P}}_{N,i}$ for the selective region and ${\text {P}}_{N,j}$ for the stopband are calculated according to

12 $$\begin{aligned} {\text {P}}_{N,i}=2 w_{\text {sel}} \begin{pmatrix} {\text {A}}_{X_N,i}-{\text {A}}_{F} \\ {\text {B}}_{X_N,i}-{\text {B}}_{F} \\ {\text {C}}_{X_N,i}-{\text {C}}_{F} \\ {\text {D}}_{X_N,i}-{\text {D}}_{F} \\ \end{pmatrix}; \ \ \ \ {\text {P}}_{N,j}=2 w_0 \begin{pmatrix} {\text {A}}_{X_N,j} \\ {\text {B}}_{X_N,j} \\ 0 \\ 0 \\ \end{pmatrix} \end{aligned}$$

The costate propagators are then backpropagated according to ${\text {P}}_k = {\text {P}}_N {\text {R}}_N \ldots {\text {R}}_{k+1}$ to calculate approximate averaged gradients by

13 $$\begin{aligned} \frac{\partial \varPhi }{\partial u_{x,k}}&=\frac{1}{2n} \sum _{i=1}^n \langle \ {\text {P}}_{k,i} | {\text {X}}_{k,i} {\text {H}}_x \rangle + \frac{1}{2m} \sum _{j=1}^m \langle \ {\text {P}}_{k,j} | {\text {X}}_{k,j} {\text {H}}_x \rangle \nonumber \\ \frac{\partial \varPhi }{\partial u_{y,k}}&=\frac{1}{2n} \sum _{i=1}^n \langle \ {\text {P}}_{k,i} | {\text {X}}_{k,i} {\text {H}}_y \rangle + \frac{1}{2m} \sum _{j=1}^m \langle \ {\text {P}}_{k,j} | {\text {X}}_{k,j} {\text {H}}_y \rangle \end{aligned}$$

where the indices *i* and *j* represent the individual calculations within the selective band and the stopbands. The overall gradients for each control and timestep *k* are then obtained by the average of the individual gradients over the different offsets in the different regions. Please note that the weights $w_{\text {sel}}$ and $w_0$ can be tailored to a specific application problem. In order to improve the performance of the pulse, the controls have to be changed by a small amount $\epsilon$ towards the global gradients according to

14 $$\begin{aligned} u_{x,k}&=u_{x,k}+\epsilon \frac{\partial \varPhi }{\partial u_{x,k}} \nonumber \\ u_{y,k}&=u_{y,k}+\epsilon \frac{\partial \varPhi }{\partial u_{y,k}} \end{aligned}$$

where $\epsilon$ can be set to a constant or optimized using a simple line minimization for each iteration. In addition, the gradient can be used directly to obtain steepest descent optimization, or by using conjugated gradients or more complex second order methods (de Fouquieres et al. 2011; Liu and Nocedal 1989; Goodwin and Kuprov 2016) for fastest possible convergence.

**Fig. 3:**
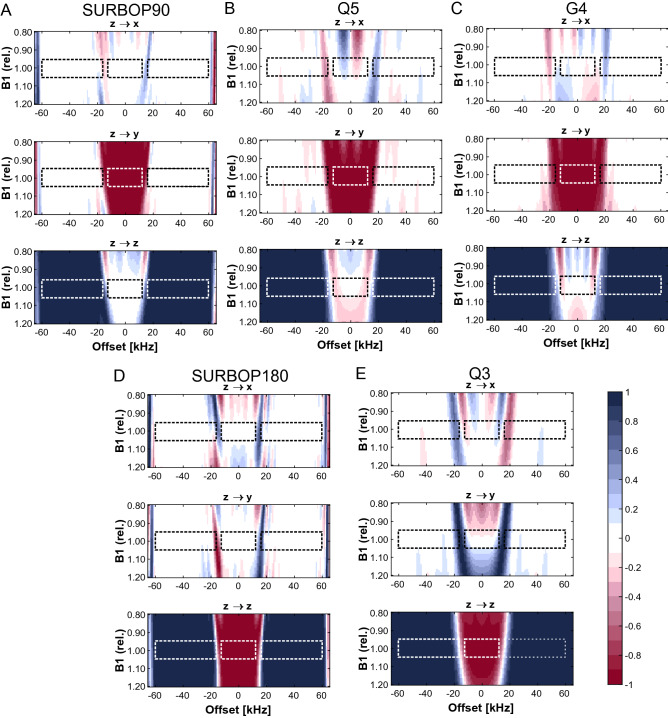
Theoretical performance of the SURBOP pulses compared to Q5/G4/Q3 Pulses: The surface plots show the values of the final magnetization components after applying SURBOP90 (**A**), Q5 (**B**), G4 (**C**), SURBOP180 (**D**), or Q3 (**E**) to initial *z*-magnetization. The dashed boxes indicate selective and stopband regions used in SURBOP optimizations. The biggest deviations from the desired behaviour are observed for Q3/Q5 pulses, especially in the selective region, when the $B_1$ field strength does not equal 1.0. G4 shows slightly smaller phase deviations than Q5 but even better results are achieved using SURBOP pulses. Their performance is essentially constant for $B_1$ deviations up to $\pm 5~\%$. The behaviour in the stopbands is mostly uniform for all pulses, but here as well, the SURBOP pulses show the best behaviour

#### Overall workflow and specific implementation

In order to optimize pulses that are robust with respect to $B_1$ inhomogeneities, five different values of maximum rf-amplitude were used corresponding to deviations of ±5 %, $\pm 2.5~\%$ and $0~\%$ from the ideal value. The frequency values chosen for *i* and *j* are equally spaced in the following ranges:

$$\omega _{i}\in [-12~{\text {kHz}},12~{\text {kHz}}]$$,

$$\omega _{j}\in [-60~{\text {kHz}},-18~{\text {kHz}}]\cup [18~{\text {kHz}},60~{\text {kHz}}]$$,

with $$i=1,2,\ldots ,30,31$$ and $$j=1,2,\ldots ,61,62$$.

The overall workflow is summarized in the following:

1. Guess initial controls $u_{x,k}$ and $u_{y,k}$.
2. Starting from ${\text {U}}_0$, calculate ${\text {X}}_{k}={\text {R}}_k{\text {R}}_{k-1}\ldots {\text {R}}_1{\text {U}}_0$ for all $k\le N$.
3. Calculate ${\text {P}}_{N}$ according to Eq. 12 and propagate it backwards in time for all $k\le N$.
4. Evaluate individual gradients (see Eq. 13).
5. Repeat steps 2–4 for all offsets in the passband and the stopbands and for different deviations from the optimal $B_1$ strength. Then calculate the average gradients of the global quality factor.
6. Update the control amplitudes according to Eq. 14.
7. Restrict controls e.g. to a maximum rf-amplitude.
8. With these new controls, go to step 2 until convergence of the optimization is reached.

#### Starting pulses

For the optimization procedure only convergence to local extrema is guaranteed, so the outcome of optimizations will depend on the starting conditions. We therefore decided to produce sets of several hundred random pulse shapes as input structures. Each shape was divided into timesteps with a duration of 0.5 ${{\mu }}$s. Random amplitudes and phases were generated for each timestep of the pulses. Pulse amplitudes were restricted to a maximum of 15 kHz.

## Pulses

In the following, the best performing pulse shapes from several thousand individual optimizatons, in which starting conditions, weighting factors $w_0$ and $w_{\text{sel}}$ as well as pulse lengths were varied systematically, are characterized in detail. The chosen pulses have a pulse length of 333.3 $\mu$s and a maximum rf-amplitude of 15.0 kHz. At each step we compare the newly derived pulses with the most commonly used standard pulses today, the Gaussian pulse cascades Q5 for universal 90$^{\circ }$ rotations and Q3 for refocusing. For some applications, a G4 point-to-point excitation pulse with lower power-requirement is sufficient. For the experimental verification we therefore considered a G4 excitation pulse with lowest rf-amplitude of the commonly used band-selective pulses and the best possible performance with respect to the power restrictions at 1.2 GHz NMR spectrometers.

### Pulse shapes

The novel pulses show significantly more amplitude and phase modulations compared to the standard pulses (see Fig. 2). The peak rf-amplitude is reached more than ten times and often maintained for several microseconds. The SURBOP180 pulse is even close to a constant amplitude pulse, giving a hint that all of the available rf-energy is needed for refocusing. The pulse shapes are not symmetric and could not be modelled by a simple mathematical formula, which is typical for pulses from GRAPE optimizations. In contrast, the shapes of the standard pulses are a lot less complex: Q5 and G4 reach values close to their peak rf-amplitudes only three times and Q3 only twice.

The phase modulations of SURBOP pulses are predominantly smooth, but include stretches with jumpy phase behaviour. In contrast, the phase of phase-alternating Q5, Q3, and G4 pulses remains constant for extended periods of time, with a few abrupt phase changes of 180$^{\circ }$.

Finally, it is important to mention that the rf-amplitude of Q5/Q3 pulses has to be scaled by a factor of approx. $\sqrt{3}=1.73$ compared to SURBOP pulses to cover the same selective region, leading to a factor 3 increase in maximum rf-power. G4 pulses need a slightly lower maximum rf-amplitude than Q3, but still 1.52 times higher than corresponding SURBOP pulses.

### Theoretical performance

To obtain a well-defined offset and $B_1$-profile of all pulses, the coherence transfer of the SURBOP pulses on equilibrium magnetization of a single spin was simulated for 141 frequency offsets within a frequency range of 130 kHz and 51 different $B_1$ field strengths (up to $\pm 20~\%$ deviation from the ideal $B_1$ value corresponding to 15.0 kHz peak rf-amplitude). The final magnetization components were plotted and compared with results from Q5, G4, and Q3 pulses (see Fig. 3).

Even though the classical pulses were applied with a higher maximum rf-amplitude (Q5/Q3: 26.0 kHz; G4: 22.7 kHz), their performance was inferior compared to SURBOP pulses. The biggest deviations from the desired behaviour are observed in the selective region, when the relative $B_1$ field strength deviates from its nominal value (1.0). G4 shows slightly smaller phase deviations than Q5 but even better results are achieved using SURBOP pulses. Their performance is essentially constant for $B_1$ deviations up to $\pm 5~\%$. The behaviour in the stopbands is generally uniform and of high quality for all pulses studied. But when looking at performance details it is again evident that the SURBOP pulses perform better, with smaller phase deviations in the optimized regions.

Additional simulations were performed to analyse, how other magnetization components are influcenced by the pulse. When starting with pure x- or y-magnetization, only universal rotation pulses (Q3/Q5/SURBOP) provide well defined offset profiles (see Supporting Information 1). When only excitation is required, G4 pulses have advantages over Q5 as they require a lower peak rf-amplitude. Therefore, in the experimental section, G4 was used as the standard 90$^{\circ }$ pulse. However, theoretical comparison with Q5 is also of great interest because the performance of the Q5 is closer to that of a UR pulse, while G4 pulses mandatorilly require time reversal for pulses rotating y-magnetisation to z-magnetisation (pulse to transfer Cα to Cβ).

### Experimental performance

**Fig. 4:**
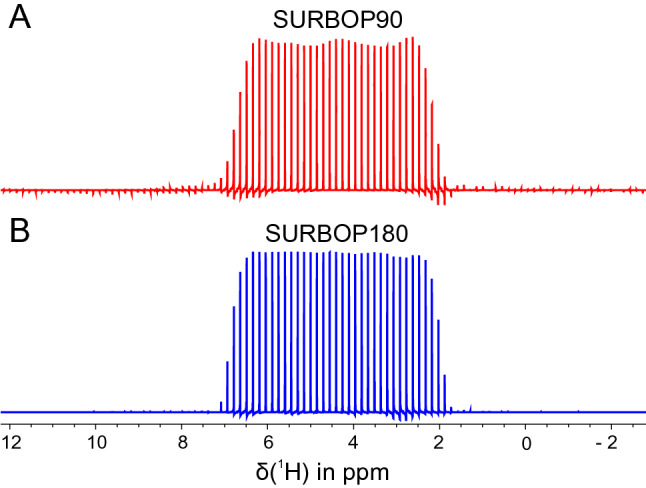
Experimentally measured offset profiles of SURBOP90 and SURBOP180: these offset profiles for SURBOP90 (**A**) and SURBOP180 pulses (**B**) were obtained by shifting the irradiation frequency in every single scan according to a frequency list of 101 offsets linearly distributed over the range of 100 kHz. Both pulses were applied with a maximum rf-amplitude of 15 kHz and a constant phase was added to the signals so that the central signal had absorptive lineshape. Fluctuations of the signal intensity are noticable in the case of SURBOP90, but deviations from ideal behaviour are generally small

The first experimental test was the aquisition of SURBOP offset profiles using a doped water sample in a Shigemi tube. For this purpose, the proton irradiation frequency was changed in every single scan according to a provided frequency list. In both cases, a frequency list with 101 offsets equally distributed over 100 kHz was defined. The pulse sequences used are provided in Supporting Information 2. Although the pulses were tested on a 400 MHz spectrometer, their rf-amplitudes were scaled to the values required at 1.2 GHz, namely 15.0 kHz. The raw data were Fourier transformed in the direct dimension and a constant phase was added so that the central signal had absorptive lineshape (zero order phase correction) before converting the data to a 1D-format. The resulting spectra are shown in Fig. 4. There are minor deviations from the desired behaviour, especially when SURBOP90 is applied. Not all isochromats in the selective region have the same phase after the pulse. The difference in peak intensity between the largest and smallest peak within the selective region amounts to 3.6 % for SURBOP180 and 5.5 % for SURBOP90. Thus, the deviations are slightly larger than observed in simulations (2.80 % and 1.53 %, respectively, in the optimized offset/B$_1$-region), which can be explained by the experimental B$_1$-inhomogeneity distribution, which significantly exceeds the specified ± 5% range. However, these effects are minor and the experimental performance is essentially comparable with the results from simulations.

**Fig. 5:**
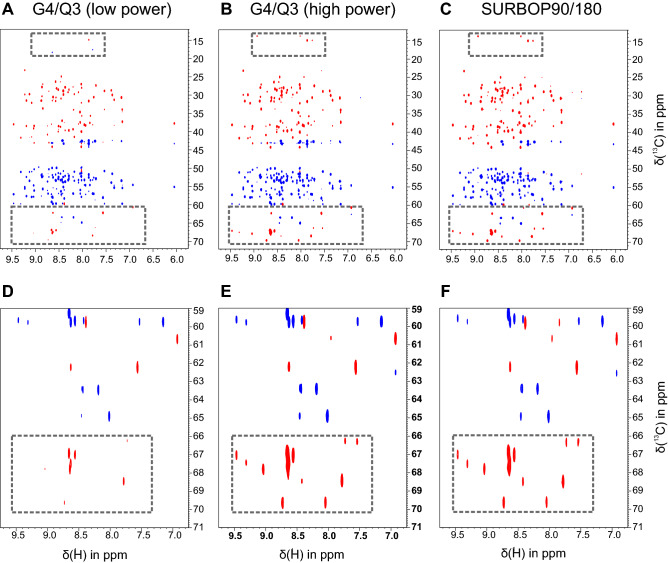
Experimental performance of SURBOP pulses compared to G4/Q3 Pulses in an HNCACB-experiment: The $^{1}$H-$^{13}$C-planes of the HNCACB experiments using low-power G4/Q3 (**A**), high-power G4/Q3 (**B**), and SURBOP pulses (**C**) are shown. The images **D**, **E** and **F** were obtained by zooming into the area between 59 and 71 ppm on the $^{13}$C axis. The spectrum recorded with low-power G4/Q3 shows less signals as well as lower signal intensities compared to the other spectra, especially in the zoomed-in area. In addition, there are undesired signals between 15 and 20 ppm, which are not present in **B** and **C**

Next, the SURBOP pulses were applied in an HNCACB experiment with a maximum rf-amplitude of 15.0 kHz to a 0.5 mm $^{13}$C, $^{15}$N labeled ubiquitin sample at 1.2 GHz $^1$H Larmor frequency.

For comparison, the same pulse sequence was applied two more times using a combination of G4 and Q3 pulses with different rf-amplitudes. First, the standard pulses were applied with peak rf-amplitudes of 22.7 and 26.0 kHz, respectively. As these values are not within the rf-power specifications of 3mm TCI and 5mm TXO CryoProbes, the experiment was also recorded with lower rf-amplitudes corresponding to 17.1 and 17.2 kHz, respectively, and adjusted pulse durations. These values allow a little buffer for salty samples with extended pulse lengths.

**Fig. 6:**
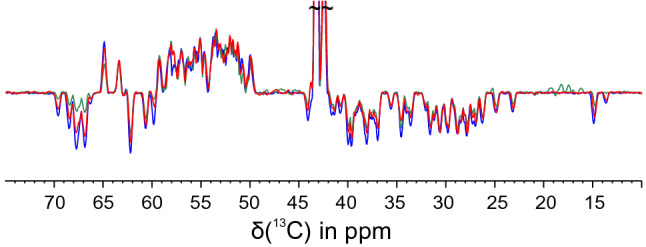
Comparison of peak intensities in HNCACB projections: In the projections of the $^{1}$H–$^{13}$C-planes of the HNCACB-experiments, the average differences in signal intensities can be evaluated most easily. The experiment using the SURBOP pulse pair (blue) shows best results. The high-power G4/Q3 projection (red) shows similar intensities for many signals, but severely reduced intensities at the edges of the selected region (e. g. for offsets > 60 ppm). The performance of the low-power G4/Q3 pair (green) is shown to be substandard, especially for negative signals

The $^{1}$H–$^{13}$C-planes resulting from the three experiments were compared in detail (Fig. 5) as well as their projections (Fig. 6). The spectra recorded with low-power G4/Q3 pulses show less signals as well as lower signal intensities compared to all other spectra, especially for $^{13}$C chemical shifts between 60 and 80 ppm, indicating an intolerable loss in S/N at the edge of the selected region. In addition, apparent artefact signals between 15 and 20 ppm were observed in this spectrum that are not present in the spectra recorded with other setups.

SURBOP pulses and high-power G4/Q3 pulses, on the other hand, deliver very similar spectra. Nevertheless, a difference in performance can be observed when comparing the projections of the $^{1}$H–$^{13}$C-plane onto the carbon chemical shift axis (see Fig. 6). These projections show the average differences in signal intensities. The experiment using the SURBOP pulse pair (blue) shows best results for signals above 60 ppm and in the range between 25 and 42 ppm and essentially equal intensities compared to the experiment with high-power G4/Q3 (red) for most other signals. The performance of the low-power G4/Q3 pair (green) is shown to be substandard, especially for signals at the edges of the aliphatic region.

At around 58 ppm, a detailed inspection reveals a single peak, for which the SURBOP peak intensity is significantly lower than intensities achieved with G4/Q3 pulses. This is caused by the summation of a positive and negative cross peak with roughly identical carbon chemical shifts. The individual signals, however, are again more intense for the SURBOP-based spectra, corroborating the results obtained for the other signals.

## Discussion

The newly introduced SURBOP 90$^\circ$ and 180$^\circ$ universal rotation pulses show significantly better performance in simulations than standard band-selective pulses like Q3/Q5 and could therefore provide an attractive and general alternative for triple resonance experiments. One of the advantages of SURBOP pulses is their compensation of B_1_-inhomogeneities, which is superb even outside the optimized range of $\pm 5\%$.

The phase modulations of SURBOP pulses are intricate and appear very jumpy at the first glance. However, a closer look shows that the curves are predominantly smooth. Despite the many phase and amplitude modulations, the pulses have been implemented on different spectrometers with modern consoles without problems. Still, further optimizations and a fundamental search for physically best pulse shapes might be useful. Eventually, slightly shorter pulse shapes might be obtained for SURBOP-90 pulses, for example. Also, a smoothing cost function or a penalty could be used in the GRAPE-type algorithm to obtain smoother pulse shapes. Alternatively, a multi-stage optimization procedure, where the optimized pulses are smoothed/varied in length and used as starting pulses in the next stage might be applied.

The pulse performance derived from experimental offset profiles is excellent, but does not fully reach the theoretical performance. This is most likely a result of damping effects that occur every time amplitude and/or phase is modified. Despite these differences between theoretical and experimental behaviour, the SURBOP pulses show better results in each triple resonance experiment acquired on a 1.2 GHz spectrometer than the standard G4/Q3 pulse pair.

For some TCI probeheads, the recommended maximum rf-amplitude is 20.833 kHz. In this case, only SURBOP pulses can be used in HNCACB and other experiments requiring selective aliphatic $^{13}$C excitation. Because SURBOP pulses have a maximum rf-amplitude of 15.0 kHz, they could be applied easily at magnetic field strengths up to 1.5 GHz (with their rf-amplitudes and duration scaled accordingly).

## Conclusion

We designed a pair of band-selective universal rotation pulses with a high ratio of selective bandwidth to maximum rf-amplitude. Therefore, these SURBOP pulses can be applied in triple resonance experiments beyond 1.0 GHz to excite the full aliphatic bandwidth, where common selective pulses like G4/Q5/Q3 start to fail. Despite their intricate amplitude and phase modulations, the novel pulses can be easily implemented on modern spectrometers.

Their excellent performance was demonstrated in simulations as well as experimentally. Our GRAPE-based algorithm can also be used in order to optimize band-selective universal rotation pulses with different parameters for other applications.

## Supplementary Information

Below is the link to the electronic supplementary material.

Electronic supplementary material 1 (PDF 735 kb)

Electronic supplementary material 2 (PDF 2459 kb)

## Data Availability

The optimized pulse shapes and data concerning SURBOP offset profiles are available at https://www.ioc.kit.edu/luy/ in the Downloads section and as part of the supplementary information. Pulses will also be available in future releases of the Bruker software TopSpin. HNCACB related data are available upon request (R. Kümmerle).
