# Supplementary material for: Band-selective universal 90° and 180° rotation pulses covering the aliphatic carbon chemical shift range for triple resonance experiments on 1.2 GHz spectrometers
Source: J Biomol NMR. 2022 Nov 24;76(5-6):185–95. doi: 10.1007/s10858-022-00404-1 (PMC9712393; doi:10.1007/s10858-022-00404-1)
Supplement: Supplementary file 1 — Electronic supplementary material 1 (PDF 735 kb) [file 10858_2022_404_MOESM1_ESM.pdf]

Selective Universal 90° and 180° Rotation Pulses Covering the Aliphatic Carbon Chemical Shift Range for Triple Resonance Experiments on 1.2 GHz Spectrometers

Supporting Information 2

*Stella Slad, Wolfgang Bermel, Rainer Kümmerle, Daniel Mathieu, and Burkhard Luy**

*Correspondence to burkhard.luy@kit.edu
Institute for Biological Interfaces 4 - Magnetic Resonance
Karlsruhe Institute of Technology (KIT)
Fritz-Haber-Weg 6, 76131 Karlsruhe

Journal of Biomolecular NMR

selzg

Pulse sequence used to obtain offset profiles of 90° shaped pulses

```
;zg2d
;avance-version (12/01/11)
;pseudo 2D sequence
;
;$CLASS=HighRes
;$DIM=2D
;$TYPE=
;$SUBTYPE=
;$COMMENT=


#include <Avance.incl>
#include <Delay.incl>


define list<frequency> fq_list=<$FQ1LIST>


1 ze
  3m fq_list:f1
2 36m
3 d1
  (p11:sp1 ph1):f1
  go=2 ph31
  30m wr #0 if #0 ze
  3m fq_list.inc
  3m fq_list:f1
  lo to 3 times td1
exit


ph1=0 2 2 0 1 3 3 1
ph31=0 2 2 0 1 3 3 1


;pl1 : f1 channel - power level for pulse (default)
;p11: f1 channel -  90 degree high power pulse
;d1 : relaxation delay; 1-5 * T1
;ns: 1
;td1: number of experiments


;$Id: $
```

selse

Pulse sequence used to obtain offset profiles of 180° shaped pulses

```
;selse2d_fq.t1.be
;avance-version (20/11/04)
;pseudo 2D sequence
;
;$CLASS=HighRes
;$DIM=2D
;$TYPE=
;$SUBTYPE=
;$COMMENT=


#include <Avance.incl>
#include <Delay.incl>
#include <Grad.incl>


define list<frequency> fq_list=<$FQ1LIST>
define list<frequency> fq_zero={sfo hz, 0.0}


"TAU1=de"
"TAU2=p1*2/PI"


"acqt0=0"
baseopt_echo


1 ze
2 36m
3 20u
  4u BLKGRAD
  d1
  50u UNBLKGRAD

  0.1u fq_zero:f1
  4u pl1:f1
  (p1 ph1):f1

  0.1u fq_list:f1

  4u
  p16:gp1
  d16
  TAU1
  (p12:sp2 ph2):f1
  TAU2
  p16:gp1
  d16
  4.1u
```

```
  go=2 ph31
  30m wr #0 if #0 ze
  3m fq_list.inc
  lo to 3 times td1
exit

ph1=0 2 2 0 1 3 3 1
ph2=1
ph31=0 2 2 0 1 3 3 1


;pl1 : f1 channel - power level for pulse (default)
;p11: f1 channel -  90 degree high power pulse
;d1 : relaxation delay; 1-5 * T1
;ns: 1
;td1: number of experiments


;for z-only gradients:
;gpz1: 31%

;use gradient files:
;gpnam1: SMSQ10.100


;$Id: $
```
