# Supplementary material for: Band-selective universal 90° and 180° rotation pulses covering the aliphatic carbon chemical shift range for triple resonance experiments on 1.2 GHz spectrometers
Source: J Biomol NMR. 2022 Nov 24;76(5-6):185–95. doi: 10.1007/s10858-022-00404-1 (PMC9712393; doi:10.1007/s10858-022-00404-1)
Supplement: Supplementary file 2 — Electronic supplementary material 2 (PDF 2459 kb) [file 10858_2022_404_MOESM2_ESM.pdf]

Selective Universal 90° and 180° Rotation Pulses Covering the Aliphatic Carbon Chemical Shift Range for Triple Resonance Experiments on 1.2 GHz Spectrometers

Supporting Information 1

*Stella Slad, Wolfgang Bermel, Rainer Kümmerle, Daniel Mathieu, and Burkhard Luy**

*Correspondence to burkhard.luy@kit.edu
Institute for Biological Interfaces 4 - Magnetic Resonance
Karlsruhe Institute of Technology (KIT)
Fritz-Haber-Weg 6, 76131 Karlsruhe

Journal of Biomolecular NMR

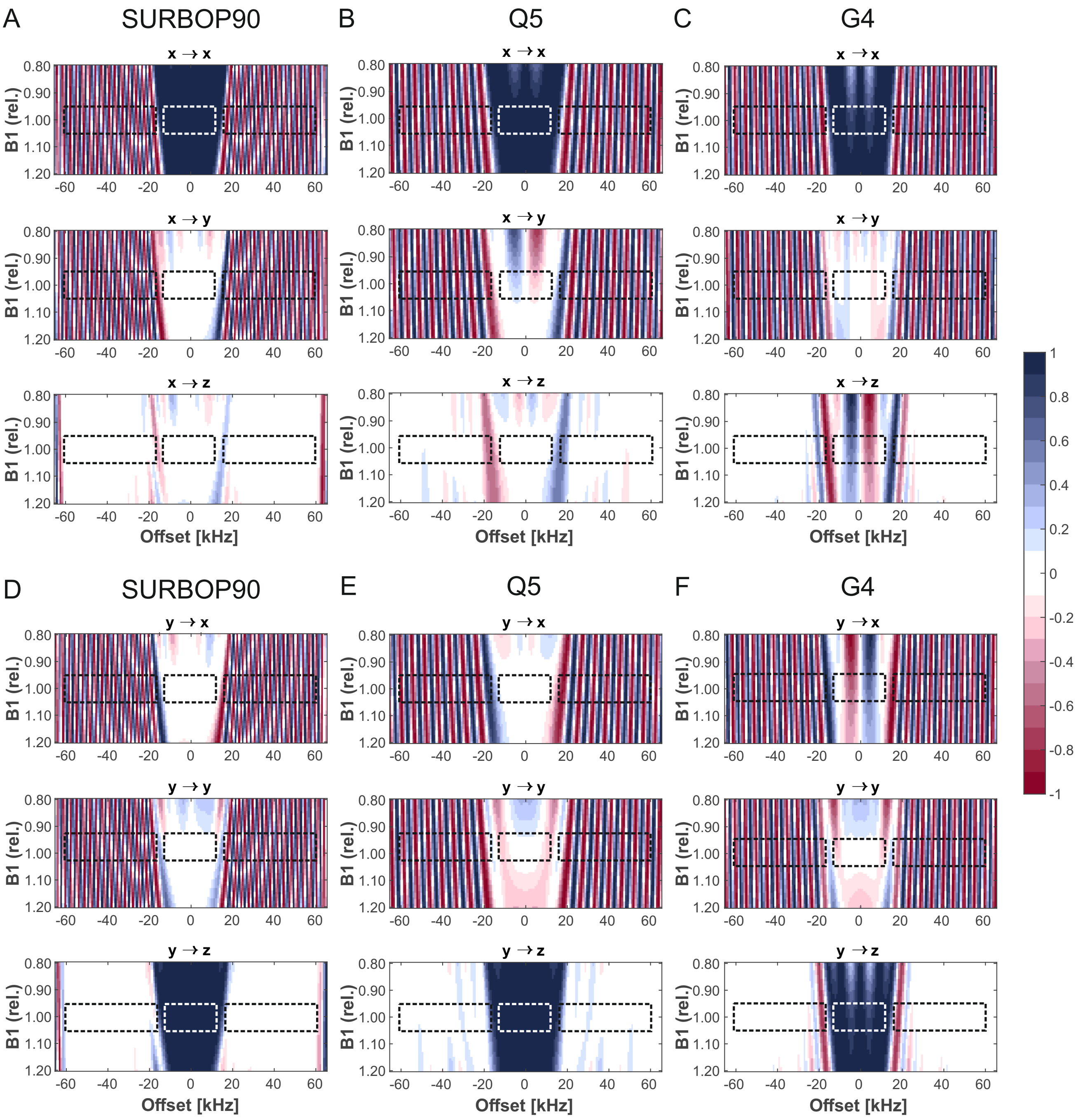

Figure 1: **Simulated effect of SURBOP90, Q5 and G4 pulses on initial *x*- and *y*-magnetization components**: The surface plots show the values of the final magnetization components after applying SURBOP90 (A, D), Q5 (B, E) or G4 (C, F) to initial *x*- or *y*-magnetization. The dashed boxes indicate selective and stopband regions used in SURBOP optimizations. The effect of the SURBOP90 pulse come closest to the desired behaviour. Q5 pulses also act similar to universal rotation pulses, however there are deviations with regards to the transfer from x- to y-magnetization. In contrast, the surface plots after applying G4 show strong phase deviations. The reason for this behaviour is that G4 pulses are optimized excitation pulses and don't act as universal rotation pulses.
